# Regulatory Framework Implementation for the Prescription of Zolpidem in France, What Impact in the Older People?

**DOI:** 10.3390/ijerph182212099

**Published:** 2021-11-18

**Authors:** Alexandra Jobert, Marion Istvan, Edouard-Jules Laforgue, Benoit Schreck, Caroline Victorri-Vigneau

**Affiliations:** 1 Nantes Université, Univ. Tours, INSERM, MethodS in Patients-Centered Outcomes and HEalth Research, SPHERE, F-44000 Nantes, France; marion.istvan@chu-nantes.fr (M.I.); edouard.laforgue@chu-nantes.fr (E.-J.L.); benoit.schreck@chu-nantes.fr (B.S.); caroline.vigneau@chu-nantes.fr (C.V.-V.); 2 CHU Nantes, Research and Innovation Department, 5 allée de l’île gloriette, CEDEX, 44093 Nantes, France; 3 CHU Nantes, Centre for Evaluation and Information on Pharmacodependence, Clinical Pharmacology Department, CEDEX, 44093 Nantes, France; 4 CHU Nantes, Addictology and Liaison-Psychiatry Department, CEDEX, 44093 Nantes, France

**Keywords:** chronic use, older people, zolpidem

## Abstract

Background: Zolpidem is one of the most prescribed hypnotic drugs. In 2001, the World Health Organization alerted a risk of pharmacodependence associated with zolpidem. The French health authority decided in 2017 to enforce security on the prescription of zolpidem to reduce those risks. The aim of our study was to evaluate the impact of regulatory framework implementation, secure prescription pad, on the prevalence and incidence of prescriptions of zolpidem according to the age. Methods: This study was based on an observational study using the French healthcare data system. Two age categories were defined: “younger” and “older” (<65 years, ≥65 years); in order to study the evolution of prevalence and incidence of zolpidem use in our two groups, two periods were defined, before and after the implementation of the measure. Results: The prevalence decreased in the younger population by 51% (4012 vs. 7948 consumers), while that of the older population decreased by 42% (4151 vs. 7282). This difference in our two groups, with a greater decrease in the younger people, is statistically significant compared to the older people. Conclusion: Our study showed that regulatory framework implementation and mandatory secure prescription pad is more effective for decreasing prevalence of zolpidem prescription for younger people compared to older people.

## 1. Introduction

Benzodiazepines (BZDs) and related drugs (Z-drugs) are used worldwide in the treatment of anxiety and sleep disorders. In its April 2017 report [1] the French National Health Product Agency (ANSM) reported that 13% of the French population consumed BZD/Z-drugs in 2015. Older people are among the strongest consumers of BZD/Z-drugs (consumption would represent 27% of all prescriptions [2]), especially in chronic use [3,4]. Excessive consumption of BZD/Z-drugs in the older people has concerning the medical consequences, such as dependence, cognitive impairment, and risk of falls. Due to age-related pharmacodynamics changes, older people are especially sensitive to sedative effects [5,6]. In addition, the summary of characteristics (SmPC) of these products specify that the dose should be reduced by half when prescribing to a patient aged 65 years or older. We also found, in a previous study, that there were signs of a slowdown in stopping treatment: among the older people with a long-term consumption studied, more of three quarters of those who tried to stop BZD/Z-drugs experienced at least one withdrawal symptom after a stopping administration [7].

Zolpidem, a Z-Drug short-acting hypnotic drug, indicated in occasional insomnia treatment is one of the most prescribed hypnotic drugs [1]. Since its marketing authorization in 1987, it has been assumed to have low potential for developing tolerance, dependence, and abuse; including in older people [8]. However, in the past several years cases of misuse, consumption at a higher dose than recommended, and abuse with zolpidem have been reported [9,10,11,12,13,14,15,16].

In 2001, the World Health Organization (WHO) alerted the risk associated with zolpidem, especially pharmacodependence, and recommended international control [17]. The Food and Drug Administration (FDA) in 2013 [18] suggested the need of dose reduction, especially for women (not age specific). A survey conducted by Nantes Centre for Evaluation and Information on Pharmacodependence (CEIP) under the oversight of the ANSM in 2002 and then in 2012 showed that zolpidem is associated with problematic drug use, such as abuse and dependence, and despite the modification of the summary of products of zolpidem, the evidence of it has a growing abuse and dependence potential over the years [9,14].

The ANSM has decided to enforce the security of prescription of zolpidem in January 2017 to reduce the risks (with an implementation of the regulation in April 2017) [19]; since then, zolpidem must be prescribed on a secure prescription pad (the number of therapeutic units per dose must be spelled out, as well as the number of doses and the dosage for the specialty; the prescription may not exceed 28 days and must be made on a specific pad with a watermark and in addition a “safety square” in which must appear the number of drugs prescribed).

The French national ZORRO study (ZOlpidem and the Reinforcement of Regulation of prescription Orders) [20], a study which aim to measure the impact of the implementation of the regulatory framework concerning the zolpidem in France, showed a decrease in prevalence of zolpidem users in general population. However, older people were not specifically studied, as it is common for them to be dependent [4]. Thus, the aim of our study was to evaluate the impact of the regulatory framework implementation on the prescriptions of zolpidem according to the age (<65 years, ≥65 years).

## 2. Materials and Methods

### 2.1. Study Oversight and Population

This work was based on an observational study [21], conducted by the pharmacology department of University Hospital of Nantes, using the French healthcare data system (Système National des Données de Santé, SNDS). The ZORRO study was approved by the French Committee of Protection of Persons (Comité de Protection des Personnes, CPP; approval reference 2018-A01070-55) and the National Data Protection Authority (Commission Nationale de l’Informatique et des Libertés, CNIL; approval reference 918201). The study was registered at www.clinicaltrials.gov (last accessed on 9 November 2021) under the reference NCT03584542.

In order to study specifically individuals aged 65 years or older compared to those under 65 years of age (according to the SmPC, which recommends a reduction in dose from the age of 65), we have included in the study all patients aged at least 18 years old in the Generalist Sample of Beneficiaries (EGB) in 2016, a representative 1/97th permanent sampling of the SNDS. The SNDS covers more than 98% of the French population (67 million people) from birth (or immigration) to death (or emigration), even in cases of change in occupation or health insurance regimen. The SNDS links several databases, among these the national claims database of the French national health insurance containing medication deliveries for outpatients (Système National d’Informations Interrégimes de l’Assurance Maladie: SNIIRAM) [22].

Variables collected in the database were: age at the inclusion, sex, the presence of a long-term illness via the registration in the list of chronic diseases scheme beneficiaries (“*Affections de Longue Durée*”: ALD) and the presence of Universal Complementary Healthcare Coverage *(“Couverture maladie universelle complémentaire”:* CMU-C) guaranteeing that low-income patients receive full reimbursement of healthcare costs and claims of zolpidem reimbursement. Two age categories were defined: “younger”, patients aged between 18 years and 64 years and “older”, patients aged 65 years and older. We identified zolpidem reimbursements in the database using the Anatomical Therapeutic Chemical (ATC) drug classification system from the World Health organization: ATC N05CF02.

In order to study the evolution of prevalence and incidence of zolpidem use in patients aged 65 years or older and those under 65 years, two periods were defined: period 1 (zolpidem use before the measure) from 1 July 2016 to 31 December 2016 and period 2 (zolpidem use from three months after the implementation of the new regulation), from 1 July 2017 to 31 December 2017. We have chosen periods a little far from the announcement of the implementation of the measure in order to avoid the analysis bias linked to the transition period. Indeed, the information from the ANSM regarding the regulatory change was published in January 2017 and became mandatory in April 2017. The choice of these two similar periods (July to December) over these 2 years, avoids a possible bias linked to the cyclic seasonality of the prescription of zolpidem.

In each of the two periods, a prevalent user of zolpidem was defined as a patient being reimbursed for zolpidem at least once and an incident episode of zolpidem use was defined as a patient receiving a first delivery of zolpidem without any prior deliveries over the preceding 6 months.

We also studied the evolution of zolpidem prescriptions in a subgroup of long-term users from the group of the oldest patient group (65 years or older) and the youngest patient group (under 65 years of age). They were defined as patients with at least one reimbursement for zolpidem per month in October, November, and December, 2016; 3 months define a chronic treatment). In this subgroup, we studied the evolution of the proportion of patients with at least one reimbursement for zolpidem per month from January 2017 to December 2017.

### 2.2. Outcome

The primary outcome was the prevalence of zolpidem users and the incidence of zolpidem episodes in the overall population according to the age categories, before and after the implementation of the secured prescription. The secondary outcome was the proportion of zolpidem users per month after the measure in the subgroup of long-term users according to age groups.

### 2.3. Statistical Analysis

All statistical analyses were performed in the oldest patient group (65 years or older) and the youngest patient group (under 65 years of age). Descriptive statistics were expressed as number and percentage for categorical variables and mean and standard deviation for continuous variables. Prevalence was calculated as the number of patients with at least one reimbursement for zolpidem out of the number of individuals in the overall population. Incidence rate was calculated as the number of zolpidem treatment initiations out of the number of total insured months (patient-month). Prevalence ratios (PR) and incidence rate ratios (IRR) and 95% confidence intervals (CI) were performed using negative binomial regression. Statistical analyses were performed using SAS 9.4 (SAS Institute, Cary, NC, USA).

## 3. Results

In total, 544,912 individuals recorded in the EGB database met the eligibility criteria from 1 July 2016 to 31 December 2016, among which 134,485 (24.7%) were aged 65 years or over and 552,308 individuals from 1 July 2017 to 31 December 2017 with 130,099 (23.6%) aged 65 years or over (Table 1). Within the two groups of populations, younger and older, patients’ characteristics were similar between the two periods for age, sex, ALD, and CMU-C status. Globally, in the young population, the mean age was approximately 42 y.o., half of the population was male, 12% had a long-term illness (ALD status), and 9% were under universal health insurance (CMU-C). In the older population, the mean age was approximately 76 y.o., 43% were male, 47% had a long-term illness, and 1% were under universal health insurance (Table 1).

### 3.1. Prevalence of Zolpidem Prescriptions

The prevalence of zolpidem users was far higher in the older population compared to the younger population before and after the measure (*p* < 0.001). In the younger population, the prevalence of zolpidem prescriptions decreased from 7948 (1.9%) zolpidem prevalent users before the measure to 4012 (1.0%) after the measure (PR = 0.49, 95% CI = 0.47–0.50). The decrease was also observed in the older population, although to a slightly lesser extent (*p* < 0.001, comparison of PR), from 7282 (5.4%) zolpidem prevalent users before the measure to 4151 (3.2%) prevalent zolpidem users after the measure (PR = 0.58, 95% CI = 0.55–0.60) (Table 1).

### 3.2. Incidence of Zolpidem Prescriptions

The incidence of zolpidem initiation decreased after the application of the measure in the younger population from 169 treatment initiations per 100,000 insured months from 1 July 2016 to 31 December 2016 to 71 treatment initiations per 100,000 insured months (IRR = 0.43, 95% CI = 0.35–0.54) and also in the older population, although again in a slightly lower decrease, from 254 to 135 treatment initiations per 100,000 insured months (IRR = 0.52, 95% CI = 0.46–0.60) (Table 1). Although this difference is not significant (*p =* 0.08 for the comparison of IRR).

**Figure 1:**
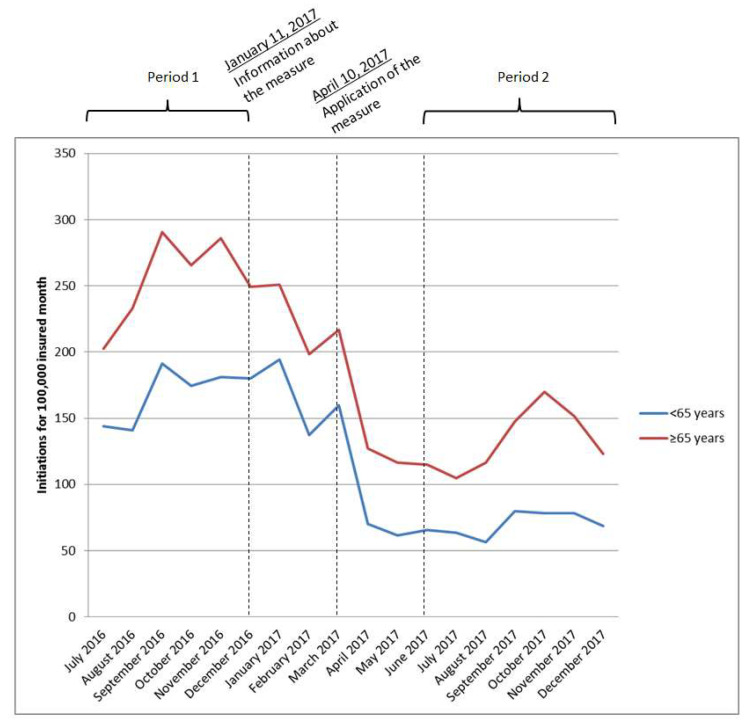
Initiation rates of zolpidem treatments according to age groups (<65 years and ≥65 years).

Treatment initiations varied on a same pattern in the younger and the older population: a first moderate decrease in January 2017 at the information from the ANSM about the measure and a second decrease in a bigger extent in April 2017 at the application of the measure. However, it should be noted that the incidence of treatment initiation seems to have begun declining earlier in the older population (in December 2016 and January 2017) than in the younger population (Figure 1).

### 3.3. Long Term Users

**Figure 2:**
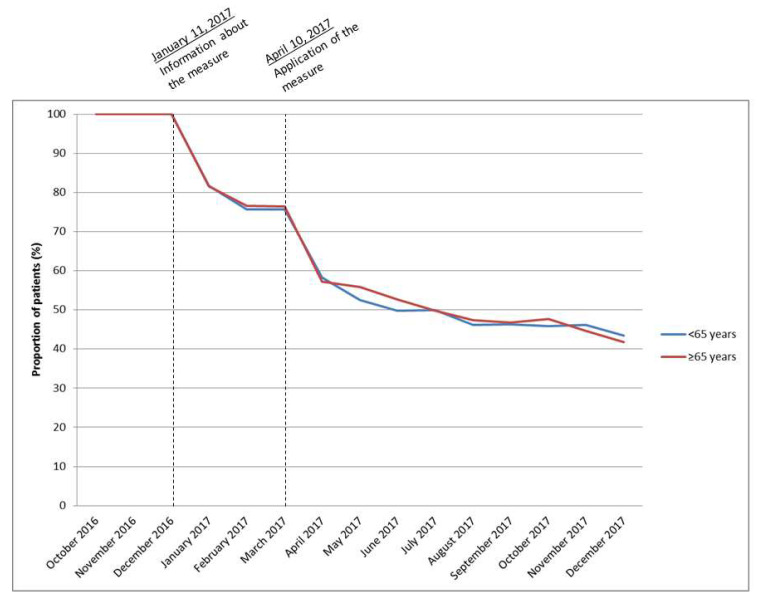
Proportion of patients with at least one reimbursement for zolpidem per month according to age groups (<65 years, ≥65 years) among long-term users (n = 2502).

In total, 2502 long-term users of zolpidem were identified before the measure, among which 1106 (44.2%) under 65 years and 1396 (55.8%) over or equal to 65 years. Among the long-term users, the proportion of patients with at least one reimbursement for zolpidem decreased similarly in the two age groups (younger and older), first at the moment of information and application of the decree and then continued to decline in the following months, respectively 43.4% (480/1106) and 41.8% (584/1396) of patients with a reimbursement for zolpidem in December 2017) (Figure 2).

## 4. Discussion

To the best of our knowledge, this work is the first that puts into perspective a particular population, the older people and to compare it to younger in the effectiveness of the measure implemented by the ANSM to reduce the consumption of zolpidem in France, which is a real public health concern in France, especially since until 2015, France ranks second in benzodiazepine consumption in Europe [1]. Our results showed a decrease in consumption of zolpidem after the change in the regulation and the requirement that it be on secure prescriptions: prevalence and incidence decreased in both younger and older people. The prevalence decreased in the younger population of 51%, while that of the older population decrease of 42%. This difference in our two groups, with a greater decrease in those under 65 years of age, is statistically significant compared to those over 65 years of age; it can therefore be assumed that the measure is more effective for those under 65 years of age. Concerning the incidence, we also showed a decrease in the younger group of 57%, again greater than that observed in the older group, 48%. Although this difference is not statistically significant, there is a trend that leads us to say that the measure seems to be even more effective in the younger group compared to the older population.

Among the 15,230 zolpidem users in the EGB, 2502 were long term users; however, we showed that when the older people represented 25% of the EGB population, they were 48% of zolpidem users and nearly 56% of long-term users. We observed a strong representation of seniors among both users and long-term users. Among long term users, the prevalence zolpidem use was similar in the two groups one year after the implementation of the measure, 43.4% among younger people and 41.8% among older people. Seniors represented only 25% of our analysis population (20% of the French population according to the National Institute of Statistics and Economic Studies (Institut national de la statistique et des études économiques INSEE) [23], yet the majority of occasional and chronic zolpidem consumers. This could be explained partly by the appearance of anxiety and depressive disorders after the cessation of professional activity [24], but also by systematic and excessive prescription by general practitioners [25].

Several countries have adopted measures to limit drug risk. Despite frequent recommendations and regulations concerning drugs associated with problematic uses (such as BZD/Z and opioid drugs), literature is scarce about the impact of these measures. To our knowledge, the specific concern of zolpidem and impact of its new regulations has not been studied outside of France. In a 2019 Cochrane database systematic review about the effects of educational and regulatory policies targeting prescribers, Suleman and Movik [26] found only two studies that met authors criteria of quality. The two studies assessed measures taken in the late 1990s. The first one [27] is an interrupted time series that evaluated change in the use of BZD (number of BZD users) by vulnerable patients in the state of New York compared to another control state from 12 months before and 24 months after a new program. The New York Medicaid program implemented a mandatory triplicate prescription programs (TPPs) requiring physicians to use multiple copy forms when ordering medicines, an intervention that was a form of surveillance of physicians’ prescribing patterns. Authors found a 48.1% relative decrease in benzodiazepine use as compared with predicted levels had the policy not been implemented, whereas there was no decrease in BZD use in the control group. Meanwhile, authors warned us of the very low certainty of evidence from their results. They also found that the greatest impact was on non-problematic BZD use consumers. Some authors warned about cases of severe withdrawal syndromes after this regulation, notably in the elderly population [28]. Another methodology was employed for the second study by Benedetto et al. [29] assessing the impact of different interventions to switch the antihistamine loratadine to fexofenadine. Pharmacy claims were analyzed before and after according to four groups of Health Maintenance Organization (mandatory lockout of loratadine in favor of fexofenadine, voluntary switch to fexofenadine promoted through letters to both physicians and members, voluntary switch promoted through letters to physicians only and no intervention). Loratadine decreased from 62.3% to 8.7% in the mandatory group whereas voluntary switch groups showed decreased of less than 10%. Next to these two studies, in Norway, flunitrazepam had an increase in its prescription safety level in 2003. According to Bramness et al. [30] who studied sales in defined daily doses of flunitrazepam per 1000 inhabitants per day, flunitrazepam sales decreased from 7.2 defined daily doses in 2002 to 3.0 in 2003. At the same time, authors mentioned a steady increase of zopiclone and zolpidem from 1999 to 2004. Thus, it is difficult to compare our results to the existent literature about regulations and their impact due to scarcity and heterogeneous methodologies. However, it seems that mandatory prescription programs aiming to reinforce security about drugs seems more effective in terms of reduction of drug exposure than information letters and/or voluntary programs but counterbalanced by switching to another drug.

In France, prescription and prolonged prescription of BZD/Z-drugs are major health concerns for older people, and previous measures have been implemented to improve safety of these prescriptions, such as limitation of the duration of the prescription (4 weeks nonrenewable for Z-drugs or 12 weeks for anxiolytic BZD). Among them, a pay for performance system towards general practitioner [31] aiming to reduce long half-life BZD prescription had failed. This measure led to an increase of short half-life BZD without decrease of mild and long-half-life BZD [32]. In addition, the French health authority (Haute Autorité de Santé, HAS, Saint-Denis, France) published recommendations [33] to help general practitioners for BZD/Z-drugs withdrawal for older people. Despite this, consumption of BZD/Z-drugs (and zolpidem among them) increased. Here, with this new regulatory framework, we see the effectiveness of the measure—even if it seems less obvious in older people. This concern raises an important point about the difficulty for older people to stop BZD/Z-drugs.

In a previous study, we identified the importance and the high stakes of the withdrawal symptoms: in older people, more than three-quarters of BZD/Z-drugs consumers who have experienced at least one stop in their consumption presented signs of withdrawal which can be an obstacle to stopping the drug [7]. We can hypothesize that the increase in sleep disorders [24], as well as the increase in comorbidities (we note a higher number of patients with an ALD status among seniors in our population), related to age, play a role in this difference in the reduction of this consumption. We can also hypothesize a change in societal behaviors; indeed, if today access to psychotherapy is easier and commonly accepted, it is possible that for people over 65, it is more difficult to take the step and consult a psychiatrist. On the other hand, we noted a greater proportion of occasional users among people under 65 years of age, for whom the measure seems to have a greater impact.

Moreover, chronic users for more than 10 years are negatively conditioned by these effects and therefore have much more difficulty stopping their treatment [7]. We can suppose that general practitioners, the prescribers of zolpidem, anticipated the measure, and as soon as the communication of its implementation (in January 2017), have targeted the patients who have a long-term consumption, indifferently of their age, in order to reduce the consumption of zolpidem for these patients specifically. This perception of risk according to the type of user (occasional vs. chronic) could explain why in our population of long-term users, no influence of age was observed.

The main strength of this study is that it was based on a representative sample of the national reimbursement database, for a drug that has only one indication: short-term treatment of severe insomnia in adults. Moreover, during the period of our study, there was no modification of zolpidem price or reimbursement and no change in supply availability. The validity of this database has been previously studied [22]. We must nevertheless note two limitations in our study. Indeed, data from EGB did not include patients living in a residential institution for dependent elderly persons; however, according to a survey conducted by the INSEE [23], 96% of senior men and 93% of senior women live at home. The second limitation concerns the selection of long-term users; we considered that, in order to be considered as a long-term user, a patient had to have a delivery every month for 3 months, which may be different from the reality (as a patient may come every 2 months to get a prescription); we assume that must be not different in the two groups.

Despite these two limitations, we can assume that this legal framework reduced the consumption of zolpidem and so should have beneficial effects, such as the reduction of side effects related to zolpidem, especially in young occasional consumers. Our results showed a decrease in the use of zolpidem, and are in agreement with those of other authors who have worked on this question [20,34,35,36]; however, we also showed that this decrease was different according to the age. For patients over 65 years old, the measure was less effective overall.

In order to complete this legal framework, it could be envisaged personalized management taking into account all co-morbidities and psycho-social factors of the patients, especially in older people, with therapeutic education tools to better inform patients about their treatment and its risks. In addition, tools for general practitioners could be useful to guide them in de-prescribing, or to reduce prescription renewals: especially shortest duration and smallest dose. Nevertheless, this corresponds to good practice and should be applied for all patients.

## 5. Conclusions

In conclusion, our study showed that the regulatory framework implementation, mandatory secure prescription pad, allows the reduction of consumption; however, this legal framework is more effective for the younger people compared to the older people. However, a regulatory measure alone cannot reduce the consumption of zolpidem, and BZD/Z-drugs in general, among seniors. Personalized management, taking into account all co-morbidities, as well as psycho-social factors, a better therapeutic education of the patient, allowing them to better knowledge of their treatments, as well as the risks related to them, the risks of pharmacodependence, seems necessary. Likewise, a tool guide for de-prescription for general practitioners could help avoiding several successive renewals, sometimes for several years, thus preventing senior patients from stopping their treatment.

## Figures and Tables

**Table 1:** Characteristics of the population before and after the measure according to age groups.

	Younger (<65 y.o.)		Older (≥65 y.o.)	
Period	1 July 2016 to 31 December 2016	1 July 2017 to 31 December 2017	1 July 2016 to 31 December 2016	1 July 2017 to 31 December 2017
Total number of patients	410,427	422,209	134,485	130,099
Age at inclusion (mean, SD)	41.6 (13.1)	41.0 (13.4)	75.9 (8.5)	75.6 (8.3)
Male (N, %)	205,293 (50.0)	211,342 (50.1)	58,518 (43.5)	56,471 (43.4)
ALD status (N, %)	49,227 (12.0)	52,914 (12.5)	63,726 (47.4)	63,489 (48.8)
CMU-C status (N, %)	36,557 (8.9)	37,089 (8.8)	1770 (1.3)	1552 (1.2)
Prevalence of zolpidem users (N, %)	7948 (1.9)	4012 (1.0)	7282 (5.4)	4151 (3.2)
Incidence of zolpidem initiations, per 100,000 insured months	169	71	254	135

Note: ALD status: long-term illness; CMU-C: Universal Complementary Healthcare Coverage.

## Data Availability

Given the risk for the privacy of patients, the data holder (French Healthcare Insurance Institution, CNAM) is preventing us from making our data set publicly available. However, all interested and qualified researchers will be able to be granted access to the EGB. The French Institute of Healthcare Data (Health Data Hub, www.health-data-hub.fr, accessed on 9 November 2021) is the contact for data requests.
